# Supplementary material for: MolPIF: a parameter interpolation flow model for molecule generation
Source: Bioinformatics. 2026 May 23;42(6):btag323. doi: 10.1093/bioinformatics/btag323 (PMC13242795; doi:10.1093/bioinformatics/btag323)
Supplement: btag323_Supplementary_Data [file btag323_supplementary_data.pdf]

Appendix A Details of MolPIF

The mechanism of PIF framework

Parameter Interpolation Flow (PIF) is a flow model that operates in the parameter space. Fig. A1 illustrates the training process of PIF. For a given type of probability distribution, its specific form is determined by its parameters. For a set of data points requiring fitting, an appropriate choice of parameters allows the construction of a Dirac distribution:

$$p(\mathbf{x} \mid \boldsymbol{\theta}(\mathbf{x}_{\text{data}})) = \delta(\mathbf{x} - \mathbf{x}_{\text{data}}) \tag{1}$$

Following the idea of FM [Lipman et al., 2022], we construct a flow to transform a simple distribution $p(\mathbf{x} \mid \boldsymbol{\theta}_{\text{prior}})$ into the desired data distribution $p(\mathbf{x} \mid \boldsymbol{\theta}(\mathbf{x}_{\text{data}}))$. Unlike conventional FM, which constructs the flow in the sample space, we instead build the flow in the parameter space:

$$p(\mathbf{x} \mid \boldsymbol{\theta}_t) = p(\mathbf{x} \mid f(t)\boldsymbol{\theta}(\mathbf{x}_{\text{data}}) + (1 - f(t))\boldsymbol{\theta}_{\text{prior}}) \tag{2}$$

Here, $t \in [0, 1]$, and $f(t)$ is a monotonic function satisfying $f(0) = 0$ and $f(1) = 1$. Thus, $\boldsymbol{\theta}_t$ satisfies $\boldsymbol{\theta}_0 = \boldsymbol{\theta}_{\text{prior}}$ and $\boldsymbol{\theta}_1 = \boldsymbol{\theta}(\mathbf{x}_{\text{data}})$. We draw samples from $p(\mathbf{x} \mid \boldsymbol{\theta}_t)$ and use them as inputs to the model, which is expected to output the parameters $\boldsymbol{\theta}(\mathbf{x}_{\text{data}})$ corresponding to the target data distribution.

To evaluate the accuracy of the predicted parameters $\hat{\boldsymbol{\theta}}$, we construct the predicted distribution parameters $\hat{\boldsymbol{\theta}}_{t+\Delta t}$ and the true interpolation distribution parameters $\boldsymbol{\theta}_{t+\Delta t}$ for the next time step $t + \Delta t$, and then compute the KL divergence between them as the loss function:

$$\hat{\boldsymbol{\theta}}_{t+\Delta t} = f(t + \Delta t)\hat{\boldsymbol{\theta}} + (1 - f(t + \Delta t))\boldsymbol{\theta}_{\text{prior}} \tag{3}$$

$$\boldsymbol{\theta}_{t+\Delta t} = f(t + \Delta t)\boldsymbol{\theta}(\mathbf{x}_{\text{data}}) + (1 - f(t + \Delta t))\boldsymbol{\theta}_{\text{prior}} \tag{4}$$

$$L_t = \mathbb{E}_{p_{\text{data}}}\left[D_{\text{KL}}\left(p(\mathbf{x} \mid \boldsymbol{\theta}_{t+\Delta t}) \,\|\, p(\mathbf{x} \mid \hat{\boldsymbol{\theta}}_{t+\Delta t})\right)\right], \quad t \in [0, 1) \tag{5}$$

Given a trained model $\boldsymbol{\Phi}$, the sampling procedure is as follows:

$$\hat{\boldsymbol{\theta}}_t \to \hat{\mathbf{m}}_t \xrightarrow{\boldsymbol{\Phi}} \hat{\boldsymbol{\theta}} \to \hat{\boldsymbol{\theta}}_{t+\Delta t} \to \cdots \tag{6}$$

where $\hat{\boldsymbol{\theta}}_t$ represents the model's prediction of $\boldsymbol{\theta}_t$, and $\hat{\mathbf{m}}_t$ is a sample drawn from the probability distribution parameterized by $\hat{\boldsymbol{\theta}}_t$. The sampling process proceeds from $t = 0$ to $t = 1$, with samples being drawn from the model's predicted distribution parameterized by $\hat{\boldsymbol{\theta}}_t$.

When a specific substructure of the data is fixed as a condition during generation, it can be provided as a conditional input, allowing the model to generate the remaining parts accordingly:

$$\hat{\boldsymbol{\theta}}_{\text{cond}} = \boldsymbol{\Phi}(\mathbf{m}_{t,\text{cond}}), \quad \mathbf{m}_{t,\text{cond}} \sim p(\mathbf{m} \mid \boldsymbol{\theta}_t, \boldsymbol{\theta}_{\text{cond}}) \tag{7}$$

Training and sampling algorithms of PIF

The detailed training and sampling algorithms of PIF are presented in Algorithm 1 and Algorithm 2, respectively.

Algorithm 1 Training procedure of PIF

Require: probability distribution $p(\mathbf{x}|\boldsymbol{\theta}) \in \mathcal{P}$, number of steps $n \in \mathbb{N}$, $\gamma \in \mathbb{R}^+$, $\boldsymbol{\theta}_{\text{prior}} \in \boldsymbol{\Theta}$, $\mathbf{x}_{\text{data}} \in \mathbb{R}^D$, neural network $\boldsymbol{\Phi}$, learning rate α

Sample $i \sim \mathcal{U}\{0, n-1\}$
$t \Leftarrow i/n$
$\boldsymbol{\theta}(\mathbf{x}_{\text{data}}) \in \{\mathbf{a} \mid p(\mathbf{x}|\mathbf{a}) = \delta(\mathbf{x} - \mathbf{x}_{\text{data}})\}$
$f(t) \Leftarrow 1 - \gamma^t$
$\boldsymbol{\theta}_t \Leftarrow f(t)\boldsymbol{\theta}(\mathbf{x}_{\text{data}}) + (1 - f(t))\boldsymbol{\theta}_{\text{prior}}$
Sample $\mathbf{m} \sim p(\mathbf{x}|\boldsymbol{\theta}_t)$
$\hat{\boldsymbol{\theta}} \Leftarrow \boldsymbol{\Phi}(\mathbf{m})$
$\Delta t \Leftarrow 1/n$
$\boldsymbol{\theta}_{t+\Delta t} \Leftarrow f(t + \Delta t)\boldsymbol{\theta}(\mathbf{x}_{\text{data}}) + (1 - f(t + \Delta t))\boldsymbol{\theta}_{\text{prior}}$
$\hat{\boldsymbol{\theta}}_{t+\Delta t} \Leftarrow f(t + \Delta t)\hat{\boldsymbol{\theta}} + (1 - f(t + \Delta t))\boldsymbol{\theta}_{\text{prior}}$
$L_t \Leftarrow D_{\text{KL}}\left(p(\mathbf{x}|\boldsymbol{\theta}_{t+\Delta t}) \,\|\, p(\mathbf{x}|\hat{\boldsymbol{\theta}}_{t+\Delta t})\right)$
$\boldsymbol{\Phi} \Leftarrow \boldsymbol{\Phi} - \alpha\nabla_{\boldsymbol{\Phi}} L_t$

Algorithm 2 Sampling procedure of PIF

Require: probability distribution $p(\mathbf{x}|\boldsymbol{\theta}) \in \mathcal{P}$, number of steps $n \in \mathbb{N}$, $\gamma \in \mathbb{R}^+$, $\boldsymbol{\theta}_{\text{prior}} \in \boldsymbol{\Theta}$, trained neural network $\boldsymbol{\Phi}$

$\boldsymbol{\theta}_0 \Leftarrow \boldsymbol{\theta}_{\text{prior}}$

for $i = 0$ **to** $n - 1$ **do**

  $t \Leftarrow i/n$

  Sample $\mathbf{m} \sim p(\mathbf{x}|\boldsymbol{\theta}_t)$

  $\hat{\boldsymbol{\theta}} \Leftarrow \boldsymbol{\Phi}(\mathbf{m})$

  $\Delta t \Leftarrow 1/n$

  $f(t) \Leftarrow 1 - \gamma^t$

  $\boldsymbol{\theta}_{t+\Delta t} \Leftarrow f(t+\Delta t)\hat{\boldsymbol{\theta}} + (1 - f(t+\Delta t))\boldsymbol{\theta}_{\text{prior}}$

end for

Sample $\mathbf{m}_1 \sim p(\mathbf{x}|\boldsymbol{\theta}_1)$

return $\mathbf{m}_1$

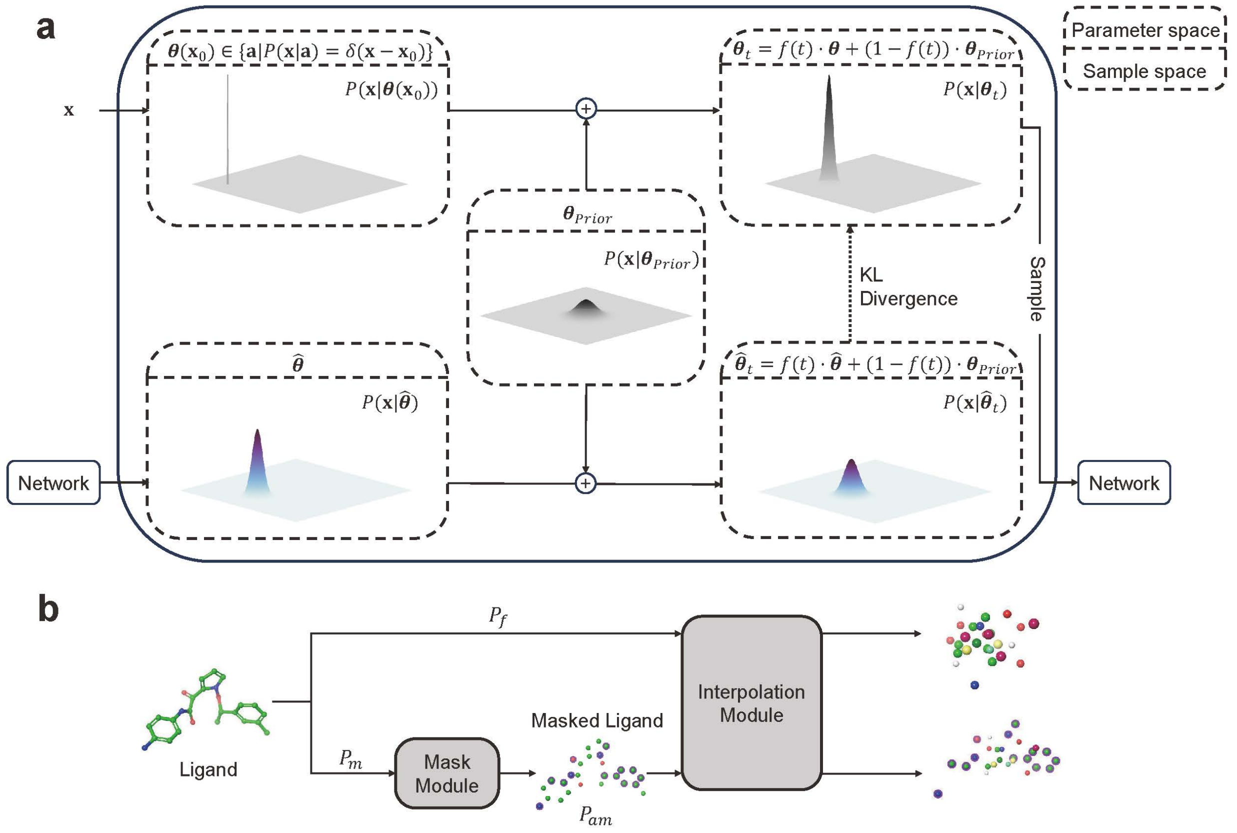

Fig. A1. Overview of the MolPIF framework. **a**, Training procedure of the PIF. At timestep t, a Dirac distribution is constructed from a variable $\mathbf{x}$, and its parameters are interpolated with the prior to obtain $\boldsymbol{\theta}_t$. The neural network predicts Dirac parameters $\hat{\boldsymbol{\theta}}_t$ from the previous output and prior interpolation. Training minimizes the KL divergence between distributions parameterized by $\boldsymbol{\theta}_t$ and $\hat{\boldsymbol{\theta}}_t$, with samples from $p(\mathbf{x} \mid \boldsymbol{\theta}_t)$ as network input. **b**, Geometry-enhanced learning strategy employed during MolPIF training. A subset of ligand atoms (probability p_m) is masked (probability p_{am}), excluded from interpolation and fixed as context.

MolPIF based on Laplace distribution

Similar to using Gaussian distributions as priors, we extend the definition of Laplace distributions by setting the parameter β to 0 for continuous-variable Dirac distributions, leading to the following reformulated computations[Soch et al., 2024]:

$$p(\mathbf{x}) = \text{La}(\mathbf{x}; \boldsymbol{\alpha}, \beta\mathbf{I}) \tag{8}$$

$$p(\mathbf{x}|\mathbf{x}_{\text{data}}) = \lim_{\beta\to 0^+} \text{La}(\mathbf{x}; \mathbf{x}_{\text{data}}, \beta\mathbf{I}), \quad \boldsymbol{\theta}_{\mathbf{x},\text{data}} = (\mathbf{x}_{\text{data}}, 0) \tag{9}$$

$$\boldsymbol{\theta}_{\mathbf{x},\text{prior}} = (\mathbf{0}, \beta_0) \tag{10}$$

$$L_{t-\Delta t,\mathbf{x}} = \mathbb{E}_{p_{\text{data}}}\left[\sum_{i=1}^{3}\left(\exp\left(-\frac{|\hat{\boldsymbol{\theta}}_{\mathbf{x}}^{(1,i)}-\boldsymbol{\theta}_{\mathbf{x}}^{(1,i)}|}{\beta_0\gamma^t}\right)+\frac{|\hat{\boldsymbol{\theta}}_{\mathbf{x}}^{(1,i)}-\boldsymbol{\theta}_{\mathbf{x}}^{(1,i)}|}{\beta_0\gamma^t}\right)\right] \tag{11}$$

Similarly, both β_0 and γ are hyperparameters.

The network structure of UniTransformer

UniTransformer is employed as the equivariant backbone for the "SE(3) NN" module. The architecture is designed to capture 3D geometric constraints through an equivariant dual-stream update mechanism: (1) Invariant features (H) are updated by integrating spatial distances (via Gaussian smearing) and chemical bond information; (2) Equivariant coordinates (X) are refined by weighting relative position vectors $(x_i - x_j)$ based on learned nodal interactions. To handle the structural complexity of protein-ligand systems, the model utilizes dynamic geometric graphs reconstructed via radius-based cutoffs and explicitly models heterogeneous interactions (e.g., Protein-Ligand vs. Ligand-Ligand) through edge-type embeddings. By adopting a "Block-Layer" hierarchy with parameter sharing, the network enables deep iterative refinement while maintaining efficiency.

Appendix B Toy data results

To evaluate our model's generalization capability, we conducted experiments on several 2D synthetic datasets. These included: (1) the swissroll and swissroll+moons datasets representing continuous distributions, and (2) sparse and dense chessboard datasets simulating discrete distributions. We replaced SLDM's moons dataset with the more complex swissroll+moons variant and introduced a dense chessboard configuration to further increase distribution complexity, challenging the model's generalization capability. All datasets contained 100,000 samples. Except for the dense chessboard experiments, hyperparameters matched those in SLDM, following `https://github.com/albarji/toy-diffusion/`; for dense chessboard, we increased training epochs to 10,000 and diffusion steps to 500 to ensure convergence (compared to 100 epochs/40 steps for swissroll and swissroll+moons, and 600 epochs/100 steps for sparse chessboard). All experiments used a 6-layer MLP with a batch size of 2,048, optimized using Adam (lr = 0.001). For SLDM, we disabled temperature control during sampling, consistent with its original implementation. As shown in Fig. B1, our model generated samples that aligned well with the original distributions, exhibiting fewer outliers and reasonable coverage compared to baseline methods. These results suggested our approach had the ability to effectively handle both continuous and discrete patterns.

Appendix C Case analysis for the performance of MolPIF in de novo molecule generation

We performed de novo generation targeting three representative binding pockets: 2V3R, 1L3L, and 6VO5 (Fig. C1). For pockets 2V3R and 1L3L (Fig. C1a–d), the top-performing candidates from 100 samples per model were analyzed. Quantitative results showed that MolPIF-generated molecules achieved superior performance across multiple metrics under fixed atom number constraints. This enhanced ligand efficiency indicates significant advantages for downstream optimization and experimental synthesis. Furthermore, we assessed MolPIF's biological relevance by targeting HAT1 (PDB: 6VO5), an enzyme critical in cancer-related acetylation processes[Wu et al., 2012]. Out of only 100 generated candidates, approximately 50% outperformed molecule H9 (Fig. C1e,f), and the vast majority showed improvement over the crystal structure reference. Notably, H9 is an experimentally validated inhibitor ($IC_{50} = 72.36\mu M$) that was originally identified from a significantly larger pool of 100,000 PocketFlow-generated molecules[Jiang et al., 2024]. These results indirectly demonstrated the efficiency of MolPIF in de novo generation of active molecules as starting points for novel targets.

Appendix D Extended data

The specific data from the experiment are presented below.

Appendix E Details of inpainting strategy

During conditional generation, MolPIF employs a "fixed-context" inpainting strategy. Unlike some diffusion-based inpainting methods where all atoms may be updated simultaneously, the coordinates and atom types of the retained substructures are kept strictly static. These fixed features serve as boundary conditions, guiding the model to generate the remaining molecular regions while preserving the original structural integrity.

Appendix F Protocol for fragment definition in the lead optimization sub-task of CBGBench

Following CBGBench, we categorize molecular components based on their functional roles in optimization tasks: Linker: A structure connecting two lead fragments (> 5 atoms each), containing at least two atoms on the shortest inter-fragment path. Fragment: The initial lead substructure in growing tasks, where the generated part maintains a 1:2 size ratio relative to the fragment. Side Chain: All terminal, non-cyclic structures attached to the core, identified via Bemis-Murcko decomposition. Scaffold: The molecular core comprising all ring systems and their connecting chains (Bemis-Murcko framework).

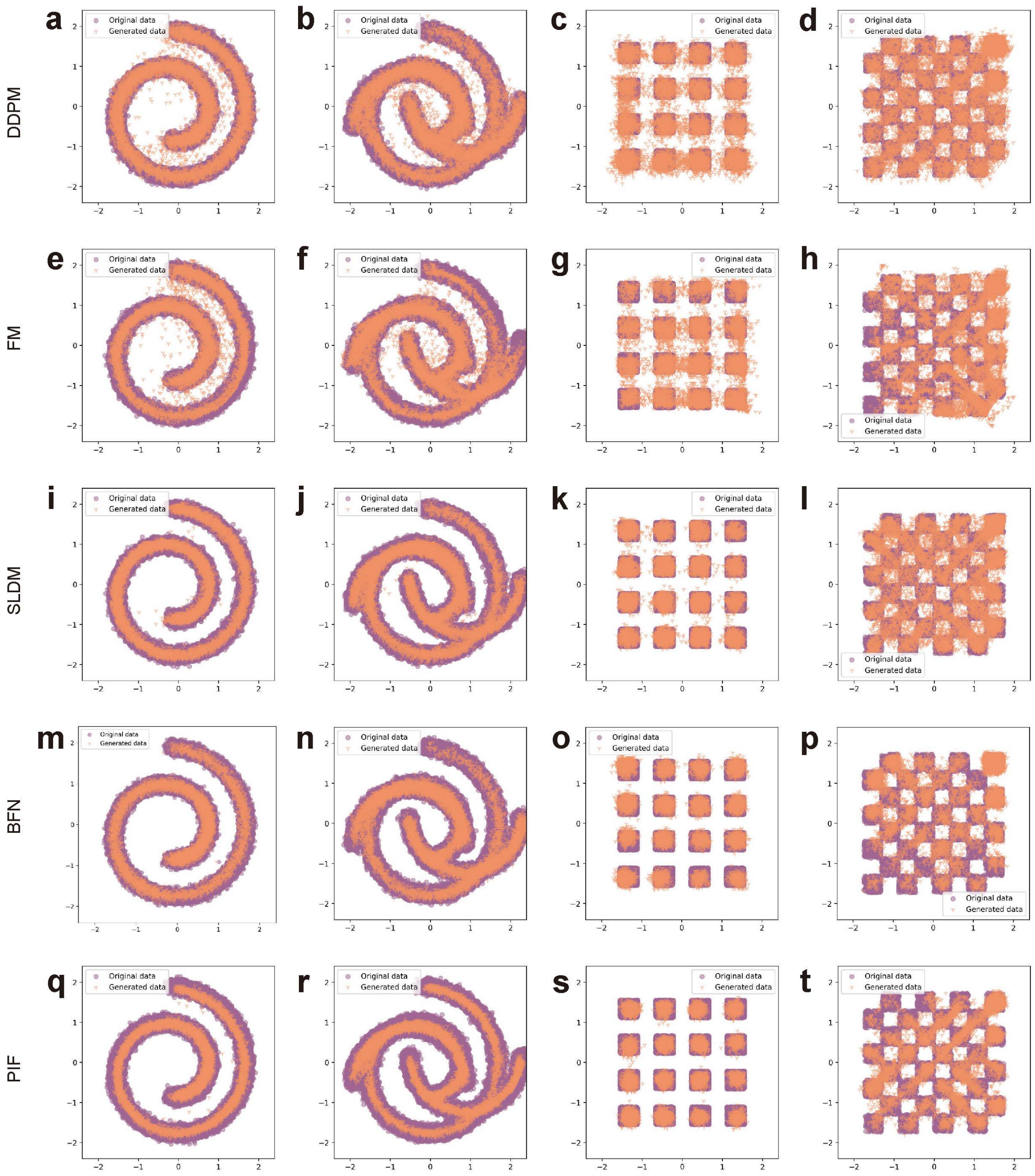

Fig. B1. Generative performance comparison on toy datasets. a-d, Performance of the DDPM on the swissroll, swissroll+moons, sparse chessboard, and dense chessboard datasets. **e-h**, Performance of the flow matching on the four datasets. **i-l**, Performance of the SLDM on the four datasets. **m-p**, Performance of the BFN on the four datasets. **q-t**, Performance of the PIF on the four datasets.

Appendix G Details of the calculation of Jensen-Shannon Divergence

The structural fidelity of generated conformations was assessed via JSD over bond lengths (JS_{BL}), angles (JS_{BA}), and torsions. The JSD is calculated by comparing the discretized probability distributions of the generated molecules against the reference set. The partitioning logic for these discrete distributions is defined as follows: Bond Lengths: Range [1.1, 1.7] Å, bin size 0.005 Å (120 bins). Bond Angles: Range [100, 140]°, bin size 0.25° (160 bins).Torsion Angles: Range [-180, 180]°, bin size 3° (120 bins).

Table D1. Ratio of different-sized rings generated by models

	3	4	5	6	7	8
AR	0.3086	0.0030	0.1556	0.4926	0.0190	0.0082
Pocket2Mol	0.0012	0.0002	0.1626	0.7983	0.0259	0.0034
TargetDiff	0.0000	0.0270	0.2971	0.4896	0.1170	0.0259
DecompDiff	0.0264	0.0391	0.3425	0.4396	0.1135	0.0178
MolCRAFT	0.0000	0.0022	0.2310	0.6986	0.0540	0.0062
MolPIF	0.0000	0.0044	0.1597	0.7688	0.0565	0.0034
MolPIF(w/o mask)	0.0000	0.0045	0.1918	0.7269	0.0614	0.0059
Test set	0.0172	0.0000	0.2961	0.6609	0.0086	0.0000

Table D2. JSD of bond lengths between reference and the molecules generated by MolCRAFT and MolPIF variants

	CC	C:C	CO	CN	C:N	OP	C:O
MolCRAFT	0.3206	0.3231	0.3536	<u>0.3010</u>	0.2497	<u>0.3410</u>	0.3339
MolPIF	0.3865	0.1978	0.4104	0.3425	**0.2068**	0.3659	0.3351
MolPIF(w/o mask)	<u>0.2885</u>	0.2257	<u>0.3475</u>	0.3161	0.2216	0.3505	0.3278
MolPIF(La)	**0.2642**	<u>0.1773</u>	**0.2527**	**0.2796**	0.2269	**0.3213**	**0.2889**
MolPIF(La w/o mask)	0.4322	**0.1555**	0.4714	0.3421	<u>0.2137</u>	0.4398	<u>0.3255</u>

Table D3. JSD of bond angles between reference and the molecules generated by MolCRAFT and MolPIF variants

	CCC	C:C:C	CCO	C:C:N	CCN
MolCRAFT	**0.3015**	<u>0.1741</u>	<u>0.3473</u>	<u>0.4508</u>	**0.3796**
MolPIF	0.3716	0.2078	0.4349	**0.4323**	0.4178
MolPIF(w/o mask)	0.3073	0.2219	0.3751	0.4509	0.4099
MolPIF(La)	<u>0.3025</u>	**0.1664**	**0.3290**	0.4670	<u>0.4058</u>
MolPIF(La w/o mask)	0.4747	0.2675	0.5551	0.4785	0.4520

Table D4. JSD of torsion angles between reference and the molecules generated by MolCRAFT and MolPIF variants

	CCCC	C:C:C:C	CCOC	CCCO
MolCRAFT	<u>0.2818</u>	**0.1555**	0.3417	0.3977
MolPIF	0.2901	0.3022	0.3417	<u>0.3911</u>
MolPIF(w/o mask)	**0.2676**	0.2667	**0.3337**	**0.3853**
MolPIF(La)	0.2953	<u>0.1734</u>	<u>0.3388</u>	0.4019
MolPIF(La w/o mask)	0.3600	0.2559	0.3555	0.3938

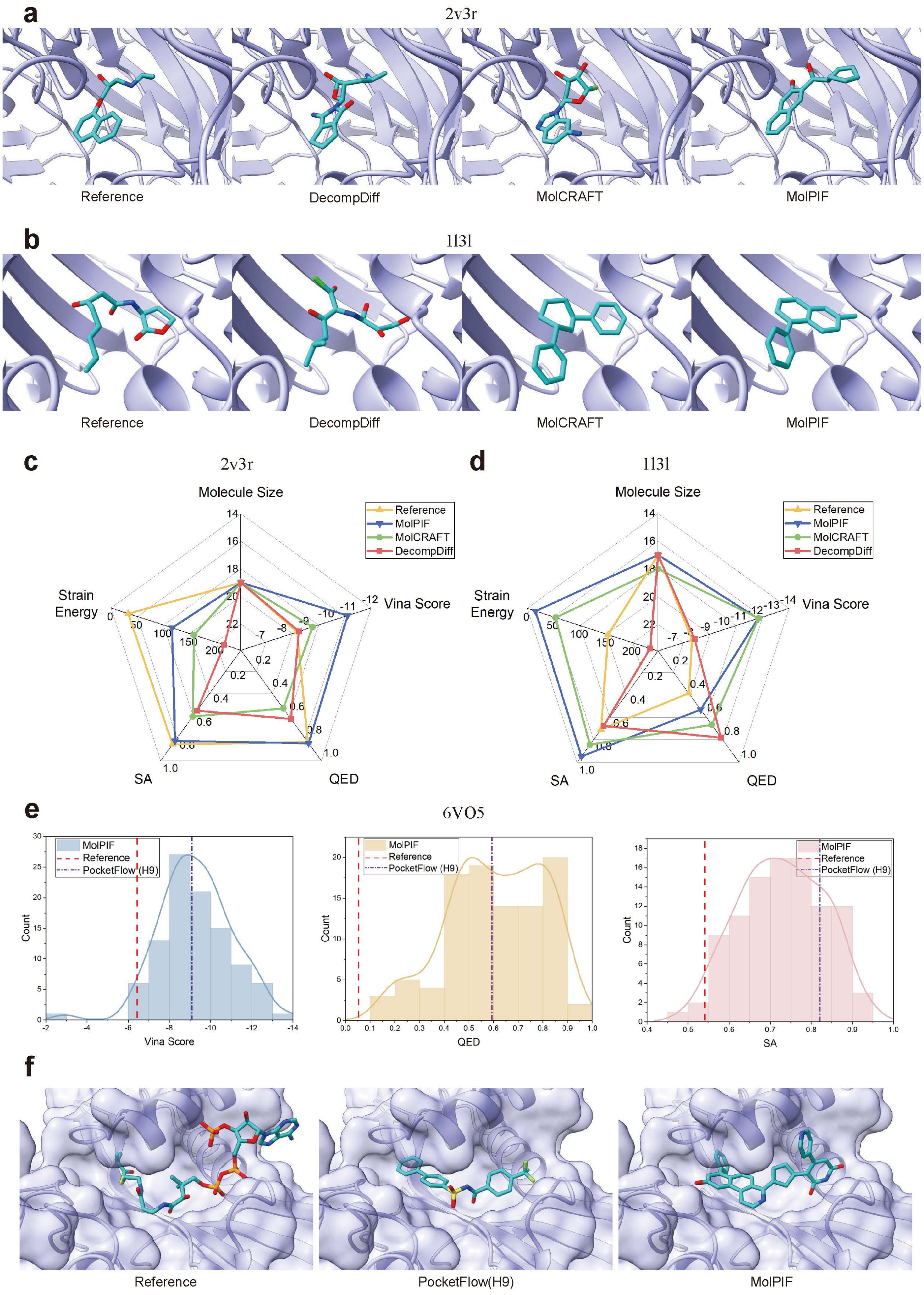

Fig. C1. Case study of generated molecules in de novo generation scenarios. a,**b**, Molecular 3D structures (DecompDiff, MolCRAFT, MolPIF) vs. reference for targets 2v3r & 1l3l. **c**,**d**, Performance of 100 molecules per method (2v3r/1l3l) across five metrics. **e**, Vina score/QED/SA distributions: MolPIF-generated (target 6VO5) vs. reference & PocketFlow molecule H9. **f**, 3D structure comparison for 6VO5 (reference, H9, MolPIF). Note: Target yield was 100 molecules per method; actual numbers may vary.

Table D5. The comparison of 100 generated molecules of MolPIF in lead optimization scenarios

	1umd					3ZCW		6KZZ	
Metric	Frag	Linker	Scaffold	Side chain	Ref.	Scaffold	Ref	Frag	Ref.
Atoms num	26	26	26	26	26	33	33	23.27	25
Mean Vina Score (↓)	-8.42	-9.40	-8.58	-8.37	-8.88	-9.29	-9.93	-8.36	-8.22
Mean Vina Min (↓)	-8.64	-9.46	-8.95	-8.75	-8.84	-9.84	-10.10	-8.60	-8.78
Mean Vina Dock (↓)	-9.02	-9.67	-9.33	-9.33	-9.39	-10.37	-10.24	-8.72	-9.12
Mean QED (↑)	0.32	0.31	0.28	0.48	0.44	0.48	0.41	0.65	0.58
Mean SA (↑)	0.58	0.57	0.58	0.56	0.66	0.67	0.80	0.81	0.88
LogP	1.08	0.76	0.62	0.35	1.72	5.32	5.11	1.80	2.52
Lipinski (↑)	4.51	4.48	4.28	4.67	5.00	4.33	4.00	4.98	5.00
SE 25% (↓)	350.39	334.74	401.24	315.30	-	252.25	-	144.90	-
SE 50% (↓)	470.68	464.40	639.84	868.76	-	332.79	-	224.68	-
SE 75% (↓)	1061.24	2152.76	2739.69	57074.30	-	563.43	-	280.16	-
SE	-	-	-	-	276.19	-	55.86	-	224.81
CR (↓)	17.54	15.06	19.60	19.49	13.00	3.78	0.00	3.85	2.00

Table D6. The proportion of MolPIF-generated molecules outperforming reference compounds in key metrics during lead optimization

	1umd				3ZCW	6KZZ
Metric	Frag	Linker	Scaffold	Side chain	Scaffold	Frag
Vina Score	0.33	0.76	0.44	0.42	0.26	0.63
Vina Min	0.42	0.84	0.57	0.54	0.40	0.43
Vina Dock	0.28	0.69	0.43	0.51	0.59	0.23
QED	0.06	0.03	0.04	0.53	0.71	0.77
SA	0.01	0.02	0.03	0.05	0.07	0.22
Lipinski	0.58	0.63	0.46	0.69	1.00	0.98
SE	0.03	0.03	0.06	0.20	0.00	0.50
CR	0.11	0.15	0.00	0.13	0.10	0.28

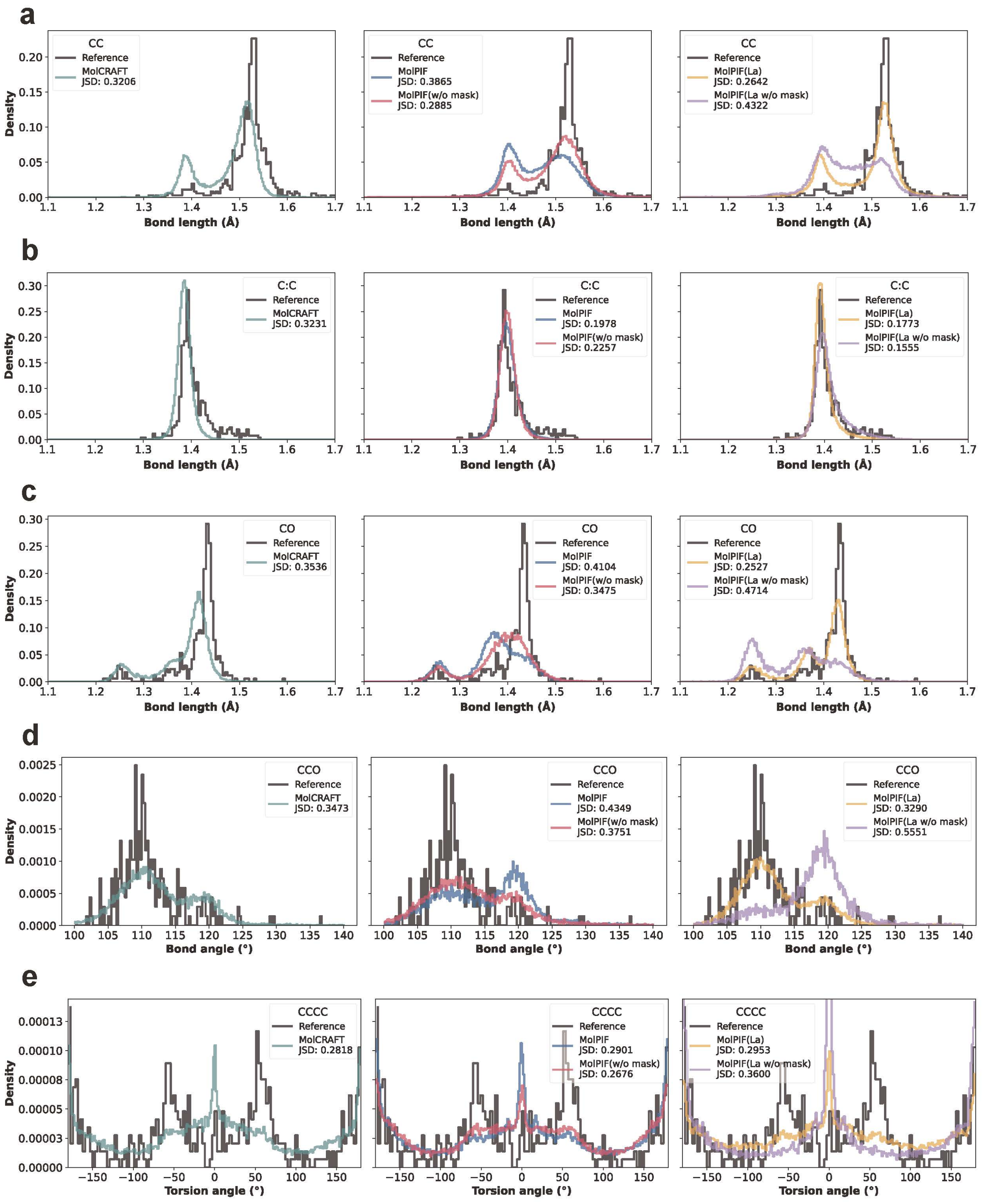

Fig. D1. Local geometry analysis of reference molecules, MolCRAFT-Generated molecules, and MolPIF Variant-Generated molecules. a-e, distributions of bond lengths (CC, C:C, CO), bond angles (CCO), and torsion angles (CCCC) in molecules generated by the models compared with the test set.

References

Y. Jiang, G. Zhang, J. You, H. Zhang, R. Yao, H. Xie, L. Zhang, Z. Xia, M. Dai, Y. Wu, et al. Pocketflow is a data-and-knowledge-driven structure-based molecular generative model. *Nature Machine Intelligence*, 6(3):326–337, 2024.

Y. Lipman, R. T. Chen, H. Ben-Hamu, M. Nickel, and M. Le. Flow matching for generative modeling. *arXiv preprint arXiv:2210.02747*, 2022.

J. Soch et al. Statproofbook/statproofbook. github. io: The book of statistical proofs, 2024.

H. Wu, N. Moshkina, J. Min, H. Zeng, J. Joshua, M.-M. Zhou, and A. N. Plotnikov. Structural basis for substrate specificity and catalysis of human histone acetyltransferase 1. *Proceedings of the National Academy of Sciences*, 109(23):8925–8930, 2012.
